# Identifying risk effectors involved in neonatal hypoglycemia occurrence

**DOI:** 10.1042/BSR20192589

**Published:** 2020-03-13

**Authors:** Tian Zhao, Qiying Liu, Man Zhou, Wei Dai, Yin Xu, Li Kuang, Yaqiong Ming, Guiyu Sun

**Affiliations:** Department of Obstetrics, Guizhou Provincial People’s Hospital, Guizhou 550002, China

**Keywords:** Clinical analysis, Hypoglycemia, Neonatal, Risk factor

## Abstract

Hypoglycemia is a common metabolic condition in neonatal period, but severe and persistent hypoglycemia can cause neurological damage and brain injury. The aim of the present study was to analyze the risk factors of neonatal hypoglycemia in clinic. A total of 135 neonatal hypoglycemia infants and 135 healthy infants were included in the present study. The differences in birth weight between neonatal hypoglycemia group and healthy control group were analyzed via *t* test. The associations between neonatal blood sugar level and relevant characteristic factors were explored using χ^2^ test. Binary logistic regression analysis was used to analyze risk factors related to the incidence of neonatal hypoglycemia. The results showed that the average birth weight was matched in neonatal hypoglycemia group and healthy control group. Neonatal blood sugar level of the infants was significantly associated with born term, birth weight, feed, gestational diabetes mellitus (GDM) and hypothermia (all *P*<0.05). Besides, logistic regression analysis showed that babies’ born term (odds ratio (OR) = 2.715, 95% confidence interval (95% CI): 1.311–5.625), birth weight (OR = 1.910, 95% CI: 1.234–2.955), improper feeding (OR = 3.165, 95% CI: 1.295–7.736) and mother’s GDM (OR = 2.184, 95% CI: 1.153–4.134) were high risk factors for neonatal hypoglycemia. The incidence of hypoglycemia in infants was significantly associated with various clinical factors. And monitoring these risk factors is one of important measures to reduce long-term neurological damage caused by neonatal hypoglycemia.

## Introduction

Hypoglycemia is a common and life-threatening complication of several diseases, such as severe malaria, bacterial sepsis, severe malnutrition and neonatal illness [1,2]. As a common metabolic condition in neonatal period, hypoglycemia reflects the process of physiological glucose metabolism and is transient in most cases [3]. The majority of neonatal hypoglycemia symptoms are hidden, and refractory hypoglycemia can lead to more severe neurological damage, and even sudden death [4–6]. Hypoglycemia usually occurs within 1–2 days after birth, especially in 6–12 h, with most of the cases being asymptomatic. In recent years, the incidence of neonatal hypoglycemia has shown an increasing trend along with the increase in birth rate and advanced technique for hypoglycemia detection. Severe and prolonged hypoglycemia can result in metal retardation, neurological deficits and recurrent seizures. In developing world, hypoglycemia remains a killer among children due to the lack of understanding on this problem.

Accumulated studies have shown that pregnancy complications, such as pregnancy-induced hypertension (PIH), gestational diabetes mellitus (GDM) and intrahepatic cholestasis (ICP), could affect perinatal situations [7–13]. Glucose plays important roles in brain metabolism and brain development in newborns. The clinical symptoms of newborns with neonatal hypoglycemia contain hypotonia, low reaction, pale, sweating, feeding difficulties and low temperature, and are often accompanied by mild to moderate disturbance of consciousness, lethargy, tremors and irritability. With the aggravation of hypoglycemia severity, newborns may show coma, epilepsy and other neurological symptoms [5]. Severe neonatal hypoglycemia can lead to poor outcome. Therefore, it is of great importance to identify factors associated with hypoglycemia so as to improve the outcomes of hypoglycemic children.

In the present study, we aimed to explore clinical factors associated with the incidence of neonatal hypoglycemia. Moreover, binary logistic regression analysis was used to analyze high risk factors for neonatal hypoglycemia.

## Materials and methods

### Study populations and general parameters

Born between October 2015 and November 2016 in Guizhou Provincial People’s Hospital, a total of 135 newborns with neonatal hypoglycemia were collected in the study, and according to 1:1 proportion, 135 healthy newborns with normal blood sugar level were randomly selected during the same period in hospital as control group. The infants in the two groups were all with gestational age ranging from 35 to 42 weeks and their birth weights were between 2000 and 4900 g.

Usually, blood glucose less than 2.6 mmol/l is an indicator for clinical intervention and treatment. In our study, venous or peripheral blood glucose less than 2.2 mmol/l (40 mg/dl) was considered as hypoglycemia. After 48–72 h of birth, the diagnosis of newborns still with hypoglycemia was considered as persistent hypoglycemia. All the newborns with hypoglycemia were regularly monitored for blood glucose, and timely treatments were performed for the cases. The main symptoms of hypoglycemia among the newborns included hypotonia, low reaction, less cry, low temperature, pale, sweating, apnea and irritability, and these symptoms would disappear after blood glucose got back to normal level, and no severe complications, such as infection or neonatal hyperbilirubinemia, were observed. Newborns with congenital metabolic diseases, endocrine disorders or hyperinsullinemia, and those with incomplete clinical data or without information from regular monitoring of blood glucose were excluded from our study.

The present study protocol was approved by the Medical Ethics Committee of Guizhou Provincial People’s Hospital. Written informed consent for sample collection was obtained before the study.

### Blood glucose determination and treatment

A total of 2–3 ml of femoral vein blood was obtained from each of the newborns within 1–2 h after birth. The blood glucose levels were measured using automatic biochemical analyzer (Backman, U.S.A.). Then the glucose levels of heel peripheral blood were monitored using micro blood glucose instrument (JNJ, U.S.A.) according to the situation.

The newborns with symptomatic hypoglycemia were immediately given 10% glucose at a dose of 2 ml/kg through intravenous injection, and then through infusion at a dose of 6–8 mg/(kg.min), thus maintaining blood glucose at 4.4–5.5 mmol/l. For asymptomatic hypoglycemia newborns, the first choice was feeding as soon as possible. If they were not able to suck, intravenous glucose infusion would be adopted for the treatment. Blood glucose levels were monitored once an hour, accompanied by the treatment of primary disease. Such monitoring would be stopped 48–72 h after blood glucose achieving normal levels.

### Statistical analysis

Data were expressed as mean ± standard deviation (SD). Statistical analyses were carried out using the software of SPSS 21.0 (SPSS, Inc., Chicago, IL, U.S.A.). The differences in birth weight between neonatal hypoglycemia group and healthy control group were analyzed with t test. The associations between neonatal blood sugar level and relevant factors were analyzed using χ^2^ test. Binary logistic regression analysis was used to analyze risk factors related to the incidence of neonatal hypoglycemia. *P*-value less than 0.05 was considered to be statistically significant.

## Results

### The characteristics of infants in the present study

A total of 135 newborns with neonatal hypoglycemia were collected as cases and 135 healthy babies as controls. The average birth weight was 3329.58 ± 630.41 g in neonatal hypoglycemia group, ranging from 2160 to 4800 g, while 3277.07 ± 464.87 g in the control group, ranging from 2000 to 4430 g. The average birth weight in the two groups was matched. The characteristics of the newborns are listed in Table 1, including gender, term, body weight, feeding, GDM, gestational hypertension and body temperature.

**Table 1:** Statistical differences of clinical parameters between neonatal hypoglycemia group and control group

Parameters	Case number	Neonatal blood sugar level		χ^2^	*P*
		Normal	Hypoglycemia		
Gender				0.134	0.714
Boy	145	74	71		
Girl	125	61	64		
Term				7.994	0.005
Full-term	227	122	105		
Premature	43	13	30		
Birth weight				15.503	<0.001
Normal	213	118	95		
LBW	28	12	16		
Macrosomia	29	5	24		
Feed				7.350	0.007
Normal	240	127	113		
Improper	30	8	22		
GDM				7.645	0.006
No	209	114	95		
Yes	61	21	40		
Gestational hypertension				0.567	0.451
No	238	121	117		
Yes	32	14	18		
Body temperature				6.595	0.010
Normal	236	125	111		
Hypothermia	34	10	24		

Abbreviation: LBW, low birth weight.

### Relationship between blood glucose levels and clinical parameters

In the present study, we analyzed the clinical characteristics both in neonatal hypoglycemia group and normal control group. As shown in Table 1, neonatal blood sugar levels were significantly associated with term (*P*=0.005), birth weight (*P*<0.001), feeding (*P*=0.007), mother’s GDM (*P*=0.006) and hypothermia (*P*=0.010). However, no significant association was found with gender or gestational hypertension (all *P*>0.05).

### Risk factors related to the incidence of neonatal hypoglycemia

To analyze risk factors correlated with neonatal hypoglycemia, we used binary logistic regression analysis. As shown in Table 2, born term (odds ratio (OR) = 2.715, 95% confidence interval (95% CI): 1.311–5.625), birth weight (OR = 1.910, 95% CI: 1.234–2.955), improper feeding (OR = 3.165, 95% CI: 1.295–7.736) and mother’s GDM (OR = 2.184, 95% CI: 1.153–4.134) were among high risk factors for the incidence of neonatal hypoglycemia (all *P*<0.05).

**Table 2:** Binary logistic regression analysis of factors contributing to neonatal hypoglycemia

Variables	β	Wald	*P*	OR	95% CI
Term	0.999	7.229	0.007	2.715	1.311–5.625
Birth weight	0.647	8.440	0.004	1.910	1.234–2.955
Feed	1.152	6.381	0.012	3.165	1.295–7.736
GDM	0.781	5.751	0.016	2.184	1.153–4.134

A two-sided *P-*value of less than 0.05 was considered statistically significant.

## Discussion

Glucose is the sole energy source for brain development during neonatal period [14]. The level of glucose in brain tissue is related to cerebral blood flow, the quantity and activity of glucose transporters in blood–brain barrier, and the available surface area. Hypoglycemia can harm multiple organs, especially for metabolically active organ, such as the brain, liver and heart [15–18]. Kirchhoff et al. [15] analyzed the long-term effects of severe hypoglycemia on brain structure and neural memory impairments in individuals with type 1 diabetes mellitus and suggested that hypoglycemia would produce permanent deleterious effects on brain structure and memory function.

Severe and persistent hypoglycemia can cause neurological damage in brain [19]. Blood tests are necessary for approximately 30% of newborns for screening neonatal hypoglycemia, and half of them will develop hypoglycemia [20]. Although clinical guidelines have offered some recommendations announcing that prophylactic measures should be taken for babies at risk of neonatal hypoglycemia, no effective measure for the prevention has been developed beyond early feeding. In the present study, we found that the average birth weight was matched in neonatal hypoglycemia group and healthy control group. The analysis results from χ^2^ test showed that neonatal hypoglycemia was significantly associated with born term, birth weight, mother’s GDM and hypothermia. Reportedly, neonatal hypoglycemia could cause many perinatal conditions. For instance, Montassir et al. [21] indicated that severe and prolonged neonatal hypoglycemia could cause cerebral lesions and other perinatal syndromes, such as hypoxia, neonatal seizure and pathological jaundice, which would exacerbate hypoglycemic brain injuries. Wong et al. [22] reported that selective posterior white matter and pulvinar edema were among the strongest predictors for clinical hypoglycemia, and that injury (36%) or watershed (32%) pattern of injury was rare in severe hypoglycemia.

In the present study, we also analyzed risk factors related to the incidence of neonatal hypoglycemia, mainly including born term, birth weight, improper feeding and mother’s GDM, and the findings were consistent with those from previous studies. For example, the study by Abu-Salah et al. [23] showed that late preterm infants had higher risk of morbidity and hospitalization than term infants, and preterm infants might suffer hypoglycemia, septicemia, feeding difficulties and significant jaundice. Staffler et al. [24] also demonstrated that preterm infants with very low birth weight were at risk of hypoglycemia. Accumulated evidences have indicated that infants born to women with GDM are at high risk for hyperinsulinism-related hypoglycemia in response to maternal hyperglycemia during pregnancy [25–27]. Hypoglycemia in infants at risk can be attenuated or cured through intervention treatments, such as oral dextrose gel or reasonable feeding [28,29].

There are still some limitations in our study. First, a large number of study subjects can improve the accuracy of the results, but the sample size in the current study was not large enough. Second, clinical parameters involved in the study analyses were limited. Third, premature birth and low birth weight are significantly correlated with neonatal hypoglycemia [30]. However, the gestational age less than 35 weeks and infants with very low birth weight were not included in the present study, In such conditions, partly premature and infants of very low birth weight were ignored, thus leading to bias in our final results. Therefore, more studies are needed to solve these issues and verify our results based on the large sample size.

In conclusion, the incidence of hypoglycemia in infants was significantly associated with born term, birth weight, improper feeding and mother’s GDM. Due to the limitations of the present study, further studies are needed to identify more effective factors for the hypoglycemia diagnosis and treatment so as to prevent brain injury.

## Abbreviations

GDM: gestational diabetes mellitus
OR: odds ratio
95% CI: 95% confidence interval
